# Quercetin: Synergistic Interaction with Antibiotics against Colistin-Resistant *Acinetobacter baumannii*

**DOI:** 10.3390/antibiotics12040739

**Published:** 2023-04-12

**Authors:** Elif Odabaş Köse, Özlem Koyuncu Özyurt, Süreyya Bilmen, Hakan Er, Cansu Kilit, Esra Aydemir

**Affiliations:** 1 Vocational School of Health Services, Akdeniz University, 07058 Antalya, Turkey; 2 Department of Medical Microbiology, Faculty of Medicine, Akdeniz University, 07070 Antalya, Turkey; 3 Department of Biophysics, Faculty of Medicine, Akdeniz University, 07070, Antalya, Turkey; 4 Department of Biology, Faculty of Science, Akdeniz University, 07070 Antalya, Turkey

**Keywords:** colistin-resistant *Acinetobacter baumannii*, quercetin, synergistic effect

## Abstract

Infections caused by resistant strains of *Acinetobacter baumannii* are now a global problem that requires the immediate development of new antimicrobial drugs. Combination therapy is one of the strategies used to solve this problem. Based on this information, the purpose of this study was to determine whether quercetin (QUE), in combination with three antibiotics, is effective against colistin-resistant *A. baumannii* strains (ColR-Ab). The effects of the combination of QUE with colistin (COL), amikacin (AMK), and meropenem (MEM) were evaluated according to the checkerboard synergy test. The combinations of QUE + COL and QUE + AMK showed synergistic activity on ColR-Ab strains with FICI values in the range of 0.1875–0.5 and 0.1875–0.2825, respectively. A 4- to 16-fold decrease in COL MIC and a 16- to 64-fold decrease in AMK MIC values were detected. Synergistic activity was confirmed by the time-kill test, and these combinations were found to be bactericidal at the end of 24 h. According to spectrophotometric measurements, the combinations of QUE + COL and QUE + AMK induced membrane damage, leading to the leakage of nucleic acids. Cell lysis and cell death were confirmed with SEM observations. The detected synergy offers an opportunity for the future development of treatment strategies for potential infections caused by ColR-Ab strains.

## 1. Introduction

*Acinetobacter baumannii*, an important nosocomial pathogen, is a gram-negative, aerobic, non-motile, and non-fermentative bacterium. This bacterium displays both high levels of natural and acquired antimicrobial resistance, as well as the ability to survive for a long time on solid and dry surfaces. *A. baumannii* frequently colonizes on abiotic surfaces (medical equipment such as ventilator tubing, humidifiers, catheters), and biotic surfaces (the skin of healthcare staff or patients). These features render it one of the most important nosocomial pathogens, easily spreading in hospital settings and contributing to higher morbidity and mortality, particularly in severely ill patients [1]. *A. baumannii* can cause a variety of severe nosocomial infections such as skin and soft tissue infections, urinary tract infections, secondary meningitis, wound infections, endocarditis, intra-abdominal abscess, and surgical site infections [2]. However, bloodstream infections and ventilator-associated pneumonia are linked to significant death rates, particularly in immunocompromised individuals [3]. Patients with an underlying disease, or who have undergone major surgical procedures are more prone to infections. Open wounds, intravascular catheters, and mechanical ventilators are ways in which *A. baumannii* can easily enter the body. Less commonly, this bacterium can cause community-acquired infections including pneumonia and bacteremia, as well as ocular, skin, and soft tissue infections, secondary meningitis, and endocarditis. Community-acquired *A. baumannii* pneumonia is more serious than nosocomial pneumonia, often severe, and has mortality rates of up to 60% [4].

Antibiotics have made it possible to treat bacterial infections that were previously untreatable and fatal [5]. However, they tend to lose their efficacy over time due to the emergence and spread of antibiotic resistance among pathogens, unlike most other drugs [6]. Infections have become more difficult or impossible to treat as a result of drug resistance, increasing the risk of the dissemination of serious infectious diseases and death. [5]. Antibiotic resistance is frequently classified as intrinsic and acquired: bacteria can be intrinsically resistant to particular antibiotics, but can also acquire resistance from their surroundings [7]. Antibiotic resistance mechanisms include the production of antibiotic-deactivating enzymes, including various classes of β-lactamases or aminoglycoside modifying enzymes, alterations in antibiotic targets, and a decrease in intracellular antibiotic concentrations, either by reducing the antibiotic’s entrance or facilitating its expulsion [8].

*A. baumannii* is a bacterium with various antimicrobial resistance mechanisms. Its critical skills include the upregulation of innate resistance mechanisms and impressively rapid acquisition of antibiotic resistance mechanisms [9]. *A. baumannii* can develop resistance to antibiotics by different mechanisms, such as the production of enzymes that degrade β-lactam antibiotics, the expression of efflux pumps, the enzymatic modification of aminoglycoside, the production of modified porins that decreases the permeability of the outer membrane, and the modification of the antibiotic target [5]. These mechanisms are responsible for the emergence of *A. baumannii* strains that are multiresistant to most antibiotics, including cephalosporin, carbapenem, aminoglycoside, and fluoroquinolone [10,11]. Carbapenem resistance in *A. baumannii* is of major concern, because carbapenems are the last line of the defense against infections caused by multidrug-resistant gram-negative bacteria. This resistance in *Acinetobacter* spp. is often associated with acquired carbapenemase production. Due to increased carbapenem resistance, second-line agents like colistin (COL) and tigecycline have been considered for the treatment of carbapenem-resistant *A. baumannii* infections [11]. Although COL was defined as a last resort for treatment, colistin-resistant *A. baumannii* strains (ColR-Ab) have been reported worldwide [12].

*A. baumannii* also has several potential virulence traits that allow it to persist in the environment, invade host cells, adhere to biotic surfaces, and escape from the human host immune system. Motility is one of the putative virulence factors of the genus. *Acinetobacter* is also resistant to disinfection and desiccation. Under dry conditions, it undergoes certain morphological changes including cell wall thickening; these changes increase its persistence on environmental surfaces. The bacterial enzyme RecA, which mediates bacterial DNA repair and resistance to desiccation, has been shown to inhibit the killing of *A. baumannii* in macrophages and contributes to death in mice [13]. Several other virulence factors have been identified. These include iron-chelating systems, lipopolysaccharides (LPS), capsular polysaccharides, phospholipases, proteases, and outer membrane porins [14]. Biofilm formation has emerged as one of the most important pathogenic features for *A. baumannii* among all virulence determinants, and rendered the organism resistant to stress factors such as desiccation, immune system clearance, and antibiotics [1]. *A. baumannii* has become one of the most critical and feared pathogens due to all of its resistance and virulence characteristics. Consequently, *A. baumannii* has been identified by the Infectious Diseases Society of America as one of the six antimicrobial-resistant pathogens that seriously threaten human health, and towards which new antibiotics are urgently required [1,15].

In the search for effective strategies, discovering new antimicrobial agents and developing combination therapies to improve the efficacy and reduce the toxicity of various drugs will help in the treatment of infections caused by resistant bacteria [16]. Plant-based products are among the agents examined as an alternative to current antibiotics used to treat multidrug-resistant bacteria [17]. Polyphenols containing polyhydroxy phytochemicals are secondary metabolites of the plant kingdom, and provide an effective defense against pathogenic aggression and UV radiation. These compounds are divided into various subclasses such as phenolic acids, tannins, flavonoids, lignans, coumarins, quinones, stilbenes, and curcuminoids according to their chemical structures. Among them, flavonoids are ubiquitous polyphenolic compounds that comprise a broad class of natural products [18]. Flavonoids have been extensively researched for their antibacterial properties, as they have traditionally been used in the prevention and treatment of various diseases such as gastrointestinal tract, urinary tract, respiratory tract, and wound infections [19].

Quercetin (QUE) (3, 3′, 4′, 5, 7-pentahydroxylflavone), is a typical flavonol-type flavonoid that is widely present in plants, including berries, apples, brassica vegetables, grapes, capers, tea, onions, and tomatoes, as well as in many seeds, nuts, flowers, bark, and leaves [20]. QUE, the most frequently studied flavonoid, has a variety of pharmacological activities including antioxidant [21], cardiovascular [22], anticancer [23], antiviral [24], neuroprotection [25], anti-inflammatory [26], and antimicrobial properties [27,28,29]. It also has GRAS (Generally Recognized As Safe) status by the United States Food and Drug Organization [30]. According to the literature, QUE, which has broad-spectrum antimicrobial properties, has also been shown to inhibit bacterial growth by working in synergy with other chemotherapeutic agents, including antibiotics [31,32,33]. It has been shown to have a good inhibitory effect on the growth of pathogenic bacteria such as *Pseudomonas aeruginosa* [31], *Escherichia coli, Klebsiella pneumoniae* [33], and *Staphylococcus aureus* [34]. Thus, QUE can be a good candidate, alone or in combination treatments, as a potent antimicrobial agent. Based on this information, we evaluated in vitro combination activities of COL, meropenem (MEM), and amikacin (AMK) with QUE against ColR-Ab strains.

## 2. Results

### 2.1. Antibacterial Susceptibility

According to broth microdilution test results, QUE exhibited antibacterial activity with Minimum Inhibitory Concentration (MIC) value of 256 µg/mL for four *A. baumannii* strains and 128 µg/mL for one. The MIC results ranged from 8 to 32 µg/mL for COL, 32 to 64 µg/mL for MEM, and 8192–16,384 µg/mL for AMK. The MICs for QUE, COL, MEM, and AMK of five *A. baumannii* strains are presented in Table 1. The MIC test results for antibiotics were evaluated based on CLSI criteria [35]. According to this, all *A. baumannii* strains were found to be resistant to the tested antibiotics.

### 2.2. Synergy Studies with QUE by Checkerboard

The activity of QUE + COL, QUE + MEM, and QUE + AMK combinations against five *A. baumannii* strains was evaluated by checkerboard synergy test. The mean FICI values observed for QUE in combination with all evaluated antibiotics across the selected strains are summarized in Table 1. According to the results, synergistic activity against the five strains was obtained with FICI values in the range of 0.1875–0.5 for the QUE + COL combination, and 0.1875–0.2825 for the QUE + AMK combination. On the other hand, it was determined that the QUE + MEM combination showed an indifferent effect against these strains with ≥0.5 FICI values.

### 2.3. Time-Kill Studies

**Figure 1:**
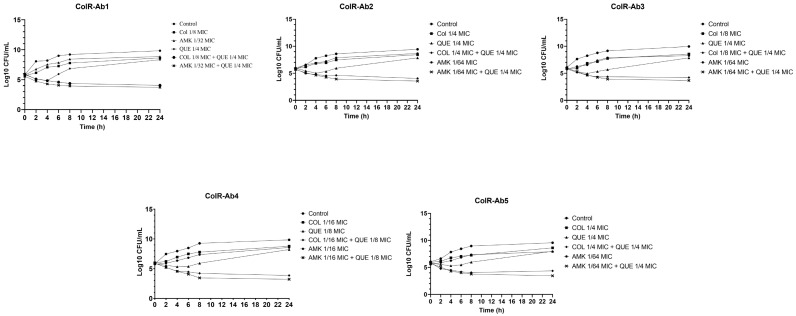
Time-kill curve analysis of all ColR-Ab strains exposed to FIC values of QUE, COL, and AMK, and synergistic concentrations of QUE + COL, and QUE + AMK.

The synergistic combinations of QUE + COL and QUE + AMK were performed using time-kill assays against five *A. baumannii* strains, which showed FICIs of ≤0.5 for the combination. These findings show that after 24 h, all strains exposed to QUE + COL and QUE + AMK had colony counts that were >2 log_10_ CFU/mL lower than those from the most active agent. Thus, the synergistic effect of both combinations was confirmed by the time-kill test. When the bactericidal activities of all agents alone and at synergistic combinations were evaluated according to the time-kill assay, none of the individual agents showed a bactericidal effect on the strains tested. When bacterial colony counts were compared to those in the control after 24 h, a decrease of <3 log_10_ CFU/mL was observed. Though, a decrease in colony counts of >3 log_10_ CFU/mL relative to the control was seen after 24 h in synergistic combinations (QUE + COL and QUE + AMK), indicating that the combination had a bactericidal impact on all strains. The results of the time-kill tests for the QUE + COL and QUE + AMK combinations on all strains are shown in the graphs in Figure 1, respectively.

### 2.4. Measuring Cell Membrane Damage

**Figure 2:**
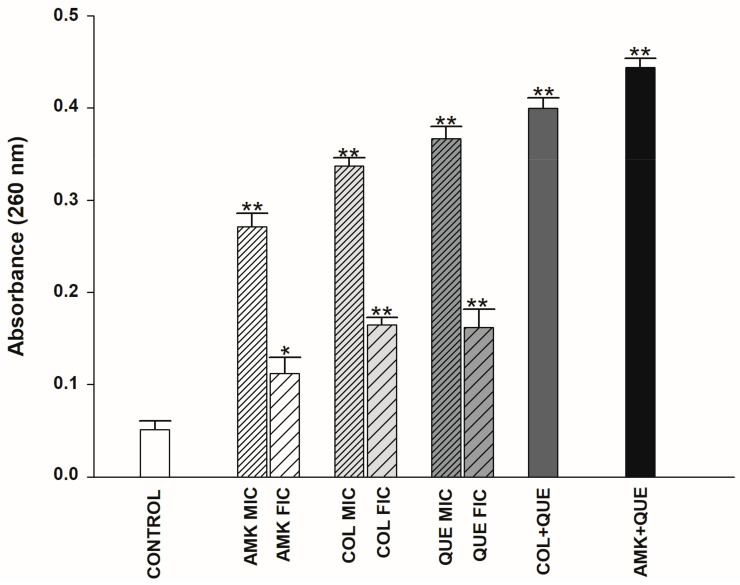
Presence of 260 nm absorbing materials in the supernatants of ColR-Ab4 strain treated with MIC and FIC values of QUE, COL, AMK, and synergistic concentrations of QUE + COL and QUE + AMK. The data are the average triplicates and * and ** significance at the levels of *p* < 0.05 and *p* < 0.01, respectively.

The absorbance of the supernatant of ColR-Ab4 strains at 260 nm was measured to determine the release of cell constituents. Figure 2 shows the resulting absorbance values when the strain was treated with COL MIC, COL FIC, AMK MIC, AMK FIC, QUE MIC, OUE FIC, QUE + AMK, and QUE + COL synergistic concentrations, respectively. Bacteria not exposed to any antimicrobial agent were used as the control. A statistically significant difference was found when the absorbance values of the agents were compared with the control. The absorbance values obtained in both synergistic combinations were higher than the values given by the agents alone. The maximum cell constituents’ release was observed when ColR-Ab4 was treated with QUE + COL combinations, showing an absorbance of 0.456.

### 2.5. Scanning Electron Microscopy (SEM) Evaluation of Morphological Alterations

**Figure 3:**
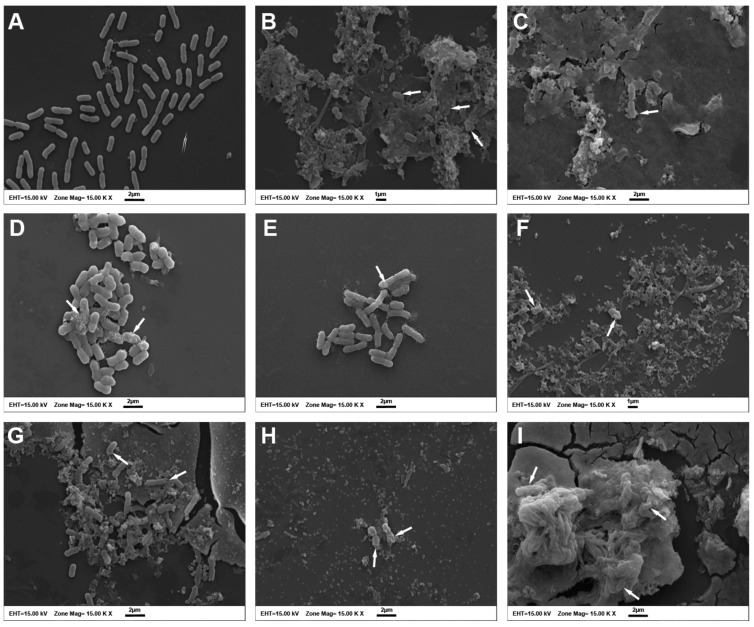
Scanning electron micrographs of ColR-Ab4 strain: (**A**) untreated; (**B**) treated with COL at MIC value; (**C**) treated with COL at FIC value; (**D**) treated with AMK at MIC value; (**E**) treated with AMK at FIC value; (**F**) treated with QUE at MIC value; (**G**) treated with QUE at FIC value; (**H**) treated with QUE + COL at the synergistic concentration; (**I**) treated with QUE + AMK at the synergistic concentration. White arrows indicate damaged bacterial cells. Scale bars, 2 µm in (**A**,**C**,**D**,**E**,**G**–**I**), and 1 µm in (**B**,**F**).

SEM analysis was used to detect morphological alterations on the surface of the ColR-Ab4 strain. The bacteria in the control group normally had rod-shaped cells with relatively smooth surfaces and intact cell membranes (Figure 3A). No changes were observed in bacteria in the FIC values of the agents, while ColR-Ab4 presented with a rough surface with numerous wrinkles in MIC values (Figure 3B–G). However, the maximum effect on bacteria was observed at synergistic concentrations of the agents. The morphology of the cell membrane was impaired; it was observed that the membranes of many cells were ruptured, and cellular contents leaked and aggregated. Most of the bacteria displayed as clumps of completely lysed cell debris (Figure 3H,I).

## 3. Discussion

Humans have been struggling with microorganisms, particularly bacteria, for a long time [36]. The healthcare community believed that with the development of antibiotics, the fight against infectious diseases had been won [37]. However, the widespread and extended use of antibiotics over time has led to the emergence of bacteria with high levels of antimicrobial resistance. This natural genetic evolution to resist antibiotics has reached paradoxical levels in the 21st century, raising antimicrobial resistance as a severe health problem with potential global implications and necessitating early intervention [36]. Antibiotic-resistant pathogen infections are generally more difficult to treat, and can repeat and cause severe morbidity and mortality [38]. According to O’Neill’s (2014) [39] estimate, if preventative measures are not adopted, there will be 10 million annual deaths from antibiotic resistance worldwide by 2050, surpassing the number of deaths from cancer, which is currently the leading cause of death. An alarming treatment gap exists between highly drug-resistant gram-negative bacteria and the options now available, according to a recent WHO assessment. In order to identify and prevent bacterial drug resistance, increasing medicinal plant resources are being explored as a result of the growing need to find novel antimicrobials [40]. Considering these data, we evaluated the combination activity of QUE with various antibiotics against ColR-Ab strains in this study. All bacterial strains selected for the study were found to be resistant to COL, MEM, and AMK. QUE alone did not show very high antimicrobial activity against these strains. However, QUE + COL and QUE + AMK combinations showed synergistic effect against five ColR-Ab strains. The combination of COL and AMK with QUE caused a decrease in the MIC values of these antibiotics. As a result of the combination of QUE + COL, MIC values decreased 4 times in two strains, 8 times in the other two strains, and 16 times in one strain. In its combination with AMK, MIC values were decreased more than COL, and a 16-fold decrease was observed in one strain, a 32-fold decrease in the other strain, and a 64-fold decrease in the remaining three strains. In other words, QUE + COL and QUE + AMK combinations caused a decrease in the resistance of *A. baumannii* strains to these antibiotics. As a result of the combination of QUE with MEM, no synergistic effect was detected. In combination, MIC values of MEM decreased 64-fold in all strains, but only a 2-fold decrease was observed in MIC values of QUE. Therefore, the activity was determined as indifferent. In the literature, no other study investigating the combined activity of COL, AMK, and MEM with QUE against ColR-Ab clinical strains was found. Consequently, this is the first study that demonstrated a combinational interaction between these agents against *A. baumannii.* However, several studies have reported the superior activity of QUE and antibiotic combinations against sensitive or resistant agents. QUE was found to increase the activity against *Streptococcus pyogenes* [41], *E. coli* [42,43], *P. aeruginosa* [31], and *K. pneumoniae* [42]. Pal and Tripathi (2019) [32] investigated the effects of QUE and MEM on carbapenem-resistant *A. baumannii* strains and found that, unlike the results of our study, this combination showed synergistic activity.

The results from the time-kill assay also showed the synergistic effect of QUE + COL and QUE + AMK against these strains. Moreover, while no single agent has a bactericidal effect, synergistic combinations have been found to have a bactericidal effect. Colony counts increased regularly in bacteria exposed to COL and AMK until the end of the incubation period. QUE alone reduced colony numbers by up to 4 h, and subsequently failed to prevent rapid regrowth of inocula. However, we found that after 24 h, COL and AMK had a bactericidal effect with the combination of QUE. These results indicate that QUE causes COL and/or AMK to acquire bactericidal activity.

Several researchers have conducted extensive study on the antibacterial properties of QUE, and have considered it as a potential treatment for a variety of pathogenic microorganisms. The antibacterial properties of QUE have been linked to its solubility and interaction with bacterial cell membranes, which are primarily impacted by the presence of hydroxyl groups in its structure [30]. Recent research has demonstrated that QUE can successfully compromise the integrity of the bacterial cell membrane, preventing bacterial development [29,32,33]. This phenomenon was also demonstrated by our experiment measuring the amount of nucleic acid released out of cells after the administration of QUE + COL and QUE + AMK synergistic combinations. Cells exposed to QUE alone had more cell membrane damage than COL. In addition, we observed the morphological changes of the bacterial cell membrane and wall after the treatment of both combinations using SEM. In fact, observation was difficult as cells exposed to the combination were extremely lysed. Since the QUE + MEM combination showed an indifferent effect in our study, we did not perform further study. However, Pal and Tripathi (2019) [32] showed with SEM observations that the combination of QUE and MEM caused membrane damage on *A. baumannii*.

Our results show that QUE significantly enhances the bactericidal capacity of COL and AMK antibiotics, and inhibits bacterial growth. Therefore, it can be suggested that the addition of QUE as a drug, in combination with COL or AMK, may reduce the overuse of antimicrobial agents and prevent the formation of bacterial resistance. QUE, as mentioned before, acts by disrupting the integrity of the bacterial membrane. COL interacts with lipid A of the lipopolysaccharide in the bacterial membrane, and modification of this structure results in acquired polymyxin resistance. AMK, an aminoglycoside antibiotic, binds to the RNA 16S of the ribosomal 30S subunit, and *A. baumannii* strains develop a resistance mechanism against this antibiotic by producing aminoglycoside-modifying enzymes [44]. The synergistic effect of the combinations of QUE with COL or AMK may be due to their different mechanisms of action on bacteria. Palaniappan and Holey (2010) [45] emphasized that the exact mechanism by which natural antimicrobials reduce antibiotic resistance is unknown, but they suggested that this is probably due to some structural changes in resistant bacteria. According to Langeveld et al. (2014) [46], most antibiotics have specific targets, and the synergy is in most cases due to the multi-target effects of the antibiotics. Similarly, Vipin et al. (2020) [31] suggest that the synergistic effect is achieved when two separate components have different mechanisms of action and may provide a higher killing effect on bacteria. The possible mechanism by which QUE increases the activity of antibiotics may be damage to the cell membrane of the bacteria. Therefore, this effect may have increased the susceptibility of *A. baumannii* to COL or AMK. However, further studies are needed to fully elucidate the mechanism by which QUE reduces antibiotic resistance.

## 4. Materials and Methods

### 4.1. Test Compounds

QUE, COL, MEM, and AMK were obtained from Sigma-Aldrich (St. Louis, MO, USA). QUE was prepared as a 2 mg/mL stock solution in ethanol and the stock solution was reconstituted before each test. All antibiotics were dissolved in sterile dH_2_O and the stock solutions of antibiotics were stored at −20 °C until assayed.

### 4.2. Bacterial Strains

Five ColR-Ab strains were obtained from the Microbiology Division of the Central Laboratory of Akdeniz University Hospital between 2019 and 2020. Blood Agar (Becton Dickinson, Franklin Lakes, NJ, USA) was used to cultivate stock solutions of *A. baumannii* strains, which were isolated from clinical samples. Matrix-Assisted Laser Desorption/Ionization Time-of-Flight (MALDI-TOF) mass spectroscopy (Bruker Daltonics, Germany) was used to identify the colonies after 18–24 h of incubation at 35 ± 2 °C. Antibiotic susceptibilities of colonies, which were identified as *A. baumannii*, were analyzed by the BD Phoenix100 automated system (Becton Dickinson, Franklin Lakes, NJ, USA) except for COL susceptibility. The automated system was evaluated according to the Clinical & Laboratory Standards Institute (CLSI). For COL sensitivity, the colistin broth disk elution method was used, and all of the colonies were resistant to COL. The antimicrobial resistance profiles of the strains are given in Table 2. Five strains that identified as COL resistant *A. baumannii* were included in the study. All isolates belonged to the patients hospitalized in the intensive care unit. Three of the isolates were trachea samples and two were pus samples. The reference strain used was *Escherichia coli* NCTC 13846. The isolates were stored at −80 °C until use and sub-cultured on blood agar for in vitro testing.

### 4.3. MIC Determination

According to the recommendations of the CLSI, the broth microdilution method was used to determine the MIC values of QUE and antibiotics [47]. Cation-adjusted Mueller Hinton Broth (MHB) (CAMHB, Merck KGaA, Darmstadt, Germany) was used in 96-well microplates to prepare the double serial dilutions of antimicrobial agents. The ranges for QUE and antibiotic concentrations were 0.25–512 and 0.0625–128 µg/mL, respectively. The bacterial suspension was added to each well after being adjusted to the 0.5 McFarland standard (final bacterial concentration: 5 × 10^5^ colony forming units [CFU]/mL). Each microdilution plate also included controls for bacterial growth (CAMHB + bacteria) and medium sterility (CAMHB). Microdilution plates were incubated at 35 ± 2 °C for 18–24 h. The MIC values were calculated by comparing the growth density in the antibiotic-containing wells to that in the control wells used in each test set. Each experiment was conducted in triplicate.

### 4.4. Checkerboard Synergy Test

The checkerboard synergy test, which is based on microdilution, was carried out to examine the combination activity of antibiotics (COL, MEM, AMK) with QUE. Using a 96-well microplate for each strain, the effectiveness of the two antimicrobial drugs in combination was evaluated. The medium used was CAMHB. The combination activity of the two agents was studied within the dilution range of 4 × MIC and 0.03125 × MIC. Decreasing concentrations of QUE was added to the wells horizontally (from column 1 to 8), while antibiotic was added vertically (from row A to G). A final inoculum of 5 × 10^5^ CFU/mL of the bacterial suspension was produced and added to each well. Additionally, each plate’s medium sterility control (CAMHB) and bacterial growth control (CAMHB + bacteria) were investigated. The plates were incubated at 35 ± 2 °C for 18–24 h. Each experiment was conducted in triplicate.

The fractional inhibition concentrations (FIC) of all antimicrobial agents were calculated in order to evaluate the results according to the following formulas:

FIC_A_ = (MIC of A in combination/MIC of A alone)

FIC_B_ = (MIC of B in combination/MIC of B alone)

FIC_index_(FICI) = FIC_A_ + FIC_B_

FICI ≤ 0.5 was considered to indicate synergism, 0.5 ≤ FICI ≤ 4 was considered to indicate indifference, and FICI > 4 was considered to indicate antagonism [48].

### 4.5. Time-Kill Assay

The time-kill test, which was studied according to the method previously defined by Moody and Knapp (2010) [49], was used to review the combinations (QUE + COL and QUE + AMK) displaying synergistic effect with QUE. In test tubes containing CAMHB, individual MIC and FIC values of the antimicrobial agents, as well as synergistic combinations of QUE + COL and QUE + AMK, were prepared for each bacterial strain. With a final bacteria density of 6 × 10^5^ CFU/mL, the bacterial suspension prepared from mid-log phase bacteria was administered to the test tubes. The sterility control was a tube with CAMHB alone, while the growth control was a tube with bacteria and CAMHB. All tubes with a total volume of 10 mL were incubated at 35 ± 2 °C. To determine the viable bacteria cell, 0.01 mL aliquots were taken from each sample at 0, 2, 4, 8, and 24 h intervals and were serially diluted in saline. The diluted samples were applied to Mueller Hinton Agar (MHA, Merck KGaA, Darmstadt, Germany) and incubated for 18–24 h at 35 ± 2 °C. In order to calculate the log of CFU/mL (log_10_ CFU/mL), the bacterial colonies between 30 and 300 CFU/mL were manually enumerated, averaged, and expressed. To confirm the results, the time-kill assay was performed in triplicate. According to the growth control, a ≥3 log_10_ reduction in CFU/mL was considered to be bactericidal activity. Synergistic activity was considered as a ≥2 log_10_ reduction in CFU/mL between the combination and its most active agents.

### 4.6. Cytoplasmic Membrane Permeability Assay

The method described by Devi et al. (2013) [50] was used to measure cell membrane damage, with minor modifications. Membrane damage measurements were conducted on a representative strain (ColR-Ab4). The bacteria were initially incubated overnight in MHB (Merck KGaA, Darmstadt, Germany) at 35 ± 2 °C. Following centrifugation of the bacterial culture at 4000× *g* for 15 min, the pellet was washed twice with PBS. The bacterial suspensions were treated with MIC (256 µg/mL QUE, 32 µg/mL COL, 8192 µg/mL AMK) and FIC (32 µg/mL QUE, 2 µg/mL COL, 512 µg/mL AMK) values of antimicrobial agents alone, and synergistic combinations of QUE + COL and QUE + AMK (32 µg/mL QUE + 2 µg/mL COL and 32 µg/mL QUE + 512 µg/mL AMK). The control was a suspension that only contained bacteria and PBS. All samples were incubated for 3 h at 35 ± 2 °C, centrifuged at 13,400× *g* for 15 min at the end of the incubation period, and the supernatant was then collected. The amount of nucleic acid released from the cytoplasm was determined by measuring the absorbance (A)_260_ of the supernatant using the Cary 60 UV–Vis spectrophotometer (Agilent Technologies, Santa Clara, CA, USA). The experiments were repeated three times.

### 4.7. Scanning Electron Microscopy

SEM examination was carried out using the Bendali et al. (2008) [51] method to see the potential impact of the antimicrobial agents alone (MIC and FIC values), and the synergistic combinations (QUE + COL and QUE + AMK) on the cell morphology of the ColR-Ab 4 strain. Bacteria were incubated at 35 ± 2 °C overnight in MH broth and then treated with MIC (256 µg/mL QUE, 32 µg/mL COL, 8192 µg/mL AMK) and FIC (32 µg/mL QUE, 2 µg/mL COL, 512 µg/mL AMK) values of individual antimicrobial agents and synergistic combinations of QUE + COL and QUE + AMK (32 µg/mL QUE + 2 µg/mL COL and 32 µg/mL QUE + 512 µg/mL AMK). Bacteria growing in MHB without antimicrobial agents were used as the control. The samples were incubated for 3 h at 35 ± 2 °C and then centrifuged for 10 min at 4000× *g*. The pellet was fixed in 2.5% glutaraldehyde at 4 °C for 2 h after being rinsed twice with PBS. The bacterial pellet was once again rinsed twice with PBS before being fixed for 1 h in 1% osmium tetroxide. The cells were then dehydrated using a graded ethanol series (30, 50, 70, 80, 90, and 100%) and rinsed twice with PBS at the end of the procedure. 100% acetone was then added instead of ethanol. The samples were finally fixed on an SEM support, coated with gold/palladium using sputtering under vacuum, and examined under a scanning electron microscope (Zeiss LEO 1430, Cambridge, UK).

### 4.8. Statistical Analyses

A professional statistics software program (Graph Pad InStat., San Diego, CA, USA) was used for the analysis of the Cytoplasmic Membrane Permeability Assay. Comparison of the results between tested groups was performed with one-way analysis of variance (ANOVA) with Dunnett’s multiple comparison post-test. The mean ± SEM was used to express all values. *p* < 0.05 was considered to be statistically significant. Sigma Plot version 10.0 (SPSS Inc., Chicago, IL, USA) software was used to create the graphs.

## 5. Conclusions

In conclusion, the combinations of QUE + COL and QUE + AMK displayed a synergistic effect on ColR-Ab strains. The effect of QUE with these antibiotics has been observed to be bactericidal by causing cell membrane damage. In this context, the combinations of QUE + COL and QUE + AMK may provide a promising new therapeutic option for the infections caused by ColR-Ab, by reducing the dosage and concentration of COL and AMK. Further studies, such as mechanism-based research, are required to reveal its therapeutic potential.

## Figures and Tables

**Table 1:** Results of the antibacterial activities of QUE, COL, AMK, and MEM, and their combination against strains of ColR-Ab.

Strains	MIC (µg/mL)				Mean FICI for QUE Combined With:		
	QUE	COL	MEM	AMK	COL	MEM	AMK
**ColR-Ab1**	128	8	64	8192	0.375 (S)	0.515 (I)	0.2825 (S)
**ColR-Ab2**	256	8	64	8192	0.5 (S)	0.515 (I)	0.2656 (S)
**ColR-Ab3**	256	32	32	8192	0.375 (S)	0.53125 (I)	0.2656 (S)
**ColR-Ab4**	256	32	64	8192	0.1875 (S)	0.515 (I)	0.1875 (S)
**ColR-Ab5**	256	8	64	16,384	0.5 (S)	0.515 (I)	0.2656 (S)
***E. coli***	128	4	≤0.0625	≤0.0625	-	-	-

S: Synergistic effect; I: indifferent effect.

**Table 2:** The antimicrobial resistance profiles for ColR-Ab strains.

Strains	MIC (µg/mL)							
	AMK	CIP	GEN	IPM	LVX	MEM	SXT	COL *
**ColR-Ab1**	>32	>1	>4	>8	>8	>8	>8/152	>4
**ColR-Ab2**	>32	>1	>4	>8	>8	>8	>8/152	>4
**ColR-Ab3**	>32	>1	>4	>8	>8	>8	>8/152	>4
**ColR-Ab4**	>32	>1	>4	>8	>8	>8	>8/152	>4
**ColR-Ab5**	>32	>1	>4	>8	>8	>8	>8/152	>4

AMK: Amikacin; CIP: Ciprofloxacin; GEN: Gentamicin; IPM: Imipenem; LVX: Levofloxacin; MEM: Meropenem; SXT: Trimethoprim/sulfamethoxazole; COL: Colistin. * Susceptibility of COL was studied by colistin broth disk elution method, while others were studied according to BD Phoenix100 system.

## Data Availability

The data presented in this study are available within the article.

## References

[1] Ayoub Moubareck C., Hammoudi Halat D. (2020). Insights into *Acinetobacter baumannii*: A Review of Microbiological, Virulence, and Resistance Traits in a Threatening Nosocomial Pathogen. Antibiotics.

[2] Almasaudi S.B. (2018). *Acinetobacter* spp. as Nosocomial Pathogens: Epidemiology and Resistance Features. Saudi J. Biol. Sci..

[3] Usmani Y., Ahmed A., Faizi S., Versiani M.A., Shamshad S., Khan S., Simjee S.U. (2021). Antimicrobial and Biofilm Inhibiting Potential of an Amide Derivative [N-(2′, 4′-Dinitrophenyl)-3β-Hydroxyurs-12-En-28-Carbonamide] of Ursolic Acid by Modulating Membrane Potential and Quorum Sensing against Colistin Resistant *Acinetobacter baumannii*. Microb. Pathog..

[4] Antunes L.C.S., Visca P., Towner K.J. (2014). *Acinetobacter baumannii*: Evolution of a Global Pathogen. Pathog. Dis..

[5] Mancuso G., Midiri A., Gerace E., Biondo C. (2021). Bacterial Antibiotic Resistance: The Most Critical Pathogens. Pathogens.

[6] Rossolini G.M., Arena F., Pecile P., Pollini S. (2014). Update on the Antibiotic Resistance Crisis. Curr. Opin. Pharmacol..

[7] Lee J.-H. (2019). Perspectives towards Antibiotic Resistance: From Molecules to Population. J. Microbiol..

[8] Kakoullis L., Papachristodoulou E., Chra P., Panos G. (2021). Mechanisms of Antibiotic Resistance in Important Gram-Positive and Gram-Negative Pathogens and Novel Antibiotic Solutions. Antibiotics.

[9] Peleg A.Y., Seifert H., Paterson D.L. (2008). *Acinetobacter baumannii:* Emergence of a Successful Pathogen. Clin. Microbiol. Rev..

[10] Howard A., O’Donoghue M., Feeney A., Sleator R.D. (2012). *Acinetobacter* *baumannii*. Virulence.

[11] Rangel K., Chagas T.P.G., De-Simone S.G. (2021). *Acinetobacter baumannii* Infections in Times of COVID-19 Pandemic. Pathogens.

[12] Lin M.-F. (2014). Antimicrobial Resistance in *Acinetobacter baumannii:* From Bench to Bedside. World J. Clin. Cases.

[13] Wong D., Nielsen T.B., Bonomo R.A., Pantapalangkoor P., Luna B., Spellberg B. (2017). Clinical and Pathophysiological Overview of *Acinetobacter* Infections: A Century of Challenges. Clin. Microbiol. Rev..

[14] Lee C.-R., Lee J.H., Park M., Park K.S., Bae I.K., Kim Y.B., Cha C.-J., Jeong B.C., Lee S.H. (2017). Biology of *Acinetobacter baumannii*: Pathogenesis, Antibiotic Resistance Mechanisms, and Prospective Treatment Options. Front. Cell. Infect. Microbiol..

[15] Qureshi Z.A., Hittle L.E., O’Hara J.A., Rivera J.I., Syed A., Shields R.K., Pasculle A.W., Ernst R.K., Doi Y. (2015). Colistin-Resistant *Acinetobacter baumannii*: Beyond Carbapenem Resistance. Clin. Infect. Dis..

[16] Lin R.-D., Chin Y.-P., Lee M.-H. (2005). Antimicrobial Activity of Antibiotics in Combination with Natural Flavonoids against Clinical Extended-Spectrumβ-Lactamase (ESBL)-Producing *Klebsiella pneumoniae*. Phytother. Res..

[17] Aleksic V., Knezevic P. (2014). Antimicrobial and Antioxidative Activity of Extracts and Essential Oils of *Myrtus communis* L.. Microbiol. Res..

[18] Mutha R.E., Tatiya A.U., Surana S.J. (2021). Flavonoids as Natural Phenolic Compounds and Their Role in Therapeutics: An Overview. Future J. Pharm. Sci..

[19] Cushnie T.P.T., Lamb A.J. (2011). Recent Advances in Understanding the Antibacterial Properties of Flavonoids. Int. J. Antimicrob. Agents.

[20] Yang D., Wang T., Long M., Li P. (2020). Quercetin: Its Main Pharmacological Activity and Potential Application in Clinical Medicine. Oxidative Med. Cell. Longev..

[21] Xu D., Hu M.-J., Wang Y.-Q., Cui Y.-L. (2019). Antioxidant Activities of Quercetin and Its Complexes for Medicinal Application. Molecules.

[22] Patel R.V., Mistry B.M., Shinde S.K., Syed R., Singh V., Shin H.-S. (2018). Therapeutic Potential of Quercetin as a Cardiovascular Agent. Eur. J. Med. Chem..

[23] Rauf A., Imran M., Khan I.A., ur-Rehman M.-, Gilani S.A., Mehmood Z., Mubarak M.S. (2018). Anticancer Potential of Quercetin: A Comprehensive Review. Phytother. Res..

[24] Di Petrillo A., Orrù G., Fais A., Fantini M.C. (2022). Quercetin and Its Derivates as Antiviral Potentials: A Comprehensive Review. Phytother. Res..

[25] Khan H., Ullah H., Aschner M., Cheang W.S., Akkol E.K. (2019). Neuroprotective Effects of Quercetin in Alzheimer’s Disease. Biomolecules.

[26] Lesjak M., Beara I., Simin N., Pintać D., Majkić T., Bekvalac K., Orčić D., Mimica-Dukić N. (2018). Antioxidant and Anti-Inflammatory Activities of Quercetin and Its Derivatives. J. Funct. Foods.

[27] He Z., Zhang X., Song Z., Li L., Chang H., Li S., Zhou W. (2020). Quercetin Inhibits Virulence Properties of *Porphyromas gingivalis* in Periodontal Disease. Sci. Rep..

[28] Roy P.K., Song M.G., Park S.Y. (2022). The Inhibitory Effect of Quercetin on Biofilm Formation of *Listeria monocytogenes* Mixed Culture and Repression of Virulence. Antioxidants.

[29] Wang S., Yao J., Zhou B., Yang J., Chaudry M.T., Wang M., Xiao F., Li Y., Yin W. (2018). Bacteriostatic Effect of Quercetin as an Antibiotic Alternative In Vivo and Its Antibacterial Mechanism In Vitro. J. Food Prot..

[30] Nguyen T.L.A., Bhattacharya D. (2022). Antimicrobial Activity of Quercetin: An Approach to Its Mechanistic Principle. Molecules.

[31] Vipin C., Saptami K., Fida F., Mujeeburahiman M., Rao S.S., Athmika, Arun A.B., Rekha P.D. (2020). Potential Synergistic Activity of Quercetin with Antibiotics against Multidrug-Resistant Clinical Strains of *Pseudomonas aeruginosa*. PLoS ONE.

[32] Pal A., Tripathi A. (2019). Quercetin Potentiates Meropenem Activity among Pathogenic Carbapenem-resistant *Pseudomonas aeruginosa* and *Acinetobacter baumannii*. J. Appl. Microbiol..

[33] Pal A., Tripathi A. (2020). Demonstration of Bactericidal and Synergistic Activity of Quercetin with Meropenem among Pathogenic Carbapenem Resistant *Escherichia coli* and *Klebsiella pneumoniae*. Microb. Pathog..

[34] Betts J.W., Sharili A.S., Phee L.M., Wareham D.W. (2015). In Vitro Activity of Epigallocatechin Gallate and Quercetin Alone and in Combination versus Clinical Isolates of Methicillin-Resistant *Staphylococcus aureus*. J. Nat. Prod..

[35] CLSI (2018). Performance Standards for Antimicrobial Susceptibility Testing.

[36] Abushaheen M.A., Muzaheed, Fatani A.J., Alosaimi M., Mansy W., George M., Acharya S., Rathod S., Divakar D.D., Jhugroo C. (2020). Antimicrobial Resistance, Mechanisms and Its Clinical Significance. Dis. Mon..

[37] Reygaert W.C. (2018). An Overview of the Antimicrobial Resistance Mechanisms of Bacteria. AIMS Microbiol..

[38] Christaki E., Marcou M., Tofarides A. (2020). Antimicrobial Resistance in Bacteria: Mechanisms, Evolution, and Persistence. J. Mol. Evol..

[39] O’Neill J. (2014). The Review on Antimicrobial Resistance. Antimicrobial Resistance: Tackling a Crisis for the Health and Wealth of Nations. https://amr-review.org/Publications.html.

[40] Tiwari P., Khare T., Shriram V., Bae H., Kumar V. (2021). Plant Synthetic Biology for Producing Potent Phyto-Antimicrobials to Combat Antimicrobial Resistance. Biotechnol. Adv..

[41] Siriwong S., Thumanu K., Hengpratom T., Eumkeb G. (2015). Synergy and Mode of Action of Ceftazidime plus Quercetin or Luteolin on *Streptococcus pyogenes*. Evid.-Based Complement. Altern. Med..

[42] Lin Y., Zhang Y., Liu S., Ye D., Chen L., Huang N., Zeng W., Liao W., Zhan Y., Zhou T. (2021). Quercetin Rejuvenates Sensitization of Colistin-Resistant *Escherichia coli* and *Klebsiella pneumoniae* Clinical Isolates to Colistin. Front. Chem..

[43] Qu S., Dai C., Shen Z., Tang Q., Wang H., Zhai B., Zhao L., Hao Z. (2019). Mechanism of Synergy Between Tetracycline and Quercetin Against Antibiotic Resistant *Escherichia coli*. Front. Microbiol..

[44] Vázquez-López R., Solano-Gálvez S.G., Juárez Vignon-Whaley J.J., Abello Vaamonde J.A., Padró Alonzo L.A., Rivera Reséndiz A., Muleiro Álvarez M., Vega López E.N., Franyuti-Kelly G., Álvarez-Hernández D.A. (2020). *Acinetobacter baumannii* Resistance: A Real Challenge for Clinicians. Antibiotics.

[45] Palaniappan K., Holley R.A. (2010). Use of Natural Antimicrobials to Increase Antibiotic Susceptibility of Drug Resistant Bacteria. Int. J. Food Microbiol..

[46] Langeveld W.T., Veldhuizen E.J.A., Burt S.A. (2014). Synergy between Essential Oil Components and Antibiotics: A Review. Crit. Rev. Microbiol..

[47] CLSI (2018). Methods for Dilution Antimicrobial Susceptibility Tests for Bacteria That Grow Aerobically.

[48] Moody J., Garcia L. (2010). Synergism Testing: Broth Microdilution Checkerboard and Broth Macrodilution Methods. Clinical Microbiology Procedures Handbook.

[49] Moody J., Knapp C., Garcia L. (2010). Tests to Assess Bactericidal Activity. Clinical Microbiology Procedures Handbook.

[50] Devi K.P., Sakthivel R., Nisha S.A., Suganthy N., Pandian S.K. (2013). Eugenol Alters the Integrity of Cell Membrane and Acts against the Nosocomial Pathogen *Proteus mirabilis*. Arch. Pharm. Res..

[51] Bendali F., Gaillard-Martinie B., Hebraud M., Sadoun D. (2008). Kinetic of Production and Mode of Action of the *Lactobacillus paracasei* subsp. *paracasei* Anti-Listerial Bacteriocin, an Algerian Isolate. LWT-Food Sci. Technol..

